# Development of Fermented Peach–Apricot Mixed Juice and Study of Its Storage Stability

**DOI:** 10.3390/foods14173128

**Published:** 2025-09-06

**Authors:** Shun Lv, Yao Zhao, Zeping Yang, Xiaolu Liu, Ruoqing Liu, Mingshan Lv, Liang Wang

**Affiliations:** 1 College of Life Science and Technology, Xinjiang University, Urumqi 830017, China; 2 College of Smart Agriculture (Research Institute), Xinjiang University, Urumqi 830017, China; 3 Department of Pharmaceutical Science, The Open University of Xinjiang, Urumqi 830063, China

**Keywords:** fermented mixed juice, lactic acid bacteria fermentation, flavour characteristics, fermentation process optimisation

## Abstract

To address the challenge of postharvest spoilage in flat peaches and white apricots, we developed fermented peach–apricot mixed juice (PAMJ) using these fruits as raw materials through multi-strain synergistic fermentation. Its fermentation processes were optimised through uniform design and single-factor experiments. The flavour characteristics of PAMJ were analysed using an electronic nose, an electronic tongue, gas chromatography–mass spectrometry (GC-MS) and sensory evaluation indices. PAMJ demonstrated optimal performance in terms of peach–apricot flavour profile, sweetness-sourness balance, and overall acceptability, achieving the highest sensory scores. Additionally, GC-MS analysis identified 116 volatile organic compounds, with PAMJ exhibiting the highest contents of terpenes and ketones. PAMJ was identified as the optimal fermentation matrix. Subsequently, response surface methodology was used to optimise its fermentation parameters. PAMJ represented a post-mixing fermentation system wherein peaches and apricots were initially mixed and subsequently fermented with a bacterial consortium comprising *Limosilactobacillus fermentum* (15%), *Lactobacillus acidophilus* (10%), *Levilactobacillus brevis* (34%), *Lacticaseibacillus paracasei* subsp. *Tolerans* (13%), *Lactiplantibacillus plantarum* subsp. *plantarum* (13%) and *Limosilactobacillus reuteri* (15%). After fermentation with an initial inoculum concentration of 5.2 × 10^6^ CFU/mL at 37 °C for 20 h, the initial soluble solid content of PAMJ increased from 16 to 16.5 °Brix, superoxide dismutase (SOD) activity increased from 250 to 295 U/mL and the number of volatile compounds (NVC) increased from 60 to 66. Furthermore, the storage stability of pasteurised PAMJ was evaluated by monitoring SOD and NVC at 5-day intervals. The data were analysed using kinetic and Arrhenius equations. The shelf life of PAMJ at 4 °C, 25 °C and 37 °C was 69, 48 and 39 days when NVC was used as the index and 99, 63 and 49 days when SOD activity was used as the index, respectively. These findings indicate that fermentation with lactic acid bacteria exerts positive effects on the quality of mixed juices, providing a novel strategy for processing speciality fruits in Xinjiang.

## 1. Introduction

Xinjiang, characterised by a unique continental arid climate and irrigation systems fed by snowmelt from the Tianshan Mountains, contains abundant speciality fruit resources. Among these fruits, Kuqa white apricots and Xinjiang flat peaches have attracted considerable attention owing to their exceptional sensory qualities and nutritional benefits. Flat peaches (*Prunus persica* L. Batsch. var. compressa Bean) and white apricots (*Prunus armeniaca* L.) belong to the *Prunus* genus within the Rosaceae family, with a cultivation history of more than a thousand years in Xinjiang, China [1]. Flat peaches are rich in various nutrients, such as chlorogenic acid, β-carotene, vitamin E and potassium, and contain unique aromatic compounds, such as γ-decalactone and linalool [2]. Kuqa white apricots thrive in the Tarim Basin, where they benefit from the combined effects of an extreme arid climate and significant day–night temperature variations. Owing to this unique environment, the apricots have an exceptionally high total soluble solid (TSS) content of 23–27 °Brix, which contributes to their pure sweet flavour [3]. Their flesh is rich in selenium, calcium, potassium, multiple B vitamins and vitamin E, earning them the local title of ‘golden fruits of the Gobi’. However, both Xinjiang flat peaches and Kuqa white apricots are climacteric fruits. Therefore, they are prone to decay after harvest, which limits their circulation period [4]. Although low-temperature storage can delay physiological deterioration, it significantly inhibits the synthesis of key volatile organic compounds (VOCs), leading to the loss of flavour compounds and degradation of nutritional quality [5]. Moreover, Xinjiang’s fruit industry faces structural challenges such as insufficient cold chain coverage, underdeveloped deep-processing technologies and a lack of branding. In recent years, many researchers have used fermentation technology to address these challenges. Through microbial biotransformation, fermentation not only extends the shelf life of processed fruits and vegetables but also enhances flavour complexity, activity of functional components and market value [6]. Consequently, this approach holds great significance for overcoming the challenges faced by Xinjiang’s fruit industry.

In Xinjiang’s fruit processing industry, fermentation techniques powered by lactic acid bacteria (LAB) have been widely adopted as a core processing method. In recent years, numerous characteristic fermented products have been developed, such as fermented juices of apples [7], tomatoes [8] and mulberries [9]. Studies on fermented apricot juice (FAJ) and fermented peach juice (FPJ) have shown that short-term fermentation with *Lactobacillus plantarum* can significantly enhance flavour and nutritional value [10,11]. However, existing studies have primarily focused on single-fruit matrices or single-strain fermentation systems. These systems have drawbacks such as limited metabolic pathways and a narrow spectrum of secondary metabolites. Studies have shown that litchi wine produced through mixed-strain fermentation contains significantly higher levels of flavour compounds than that produced through fermentation with a single yeast strain [12]. This difference results from metabolic complementarity and competition among strains. These interactions collectively promote the production of secondary metabolites while enhancing the flavour complexity and functional activity of fruit juices. Flat peaches are rich in flavour compounds, while white apricots contain high levels of fermentable sugars. This natural balance may provide an excellent biological foundation for their co-fermentation with mixed LAB strains. However, no studies have systematically investigated whether this co-fermentation can stimulate the metabolic activity of mixed strains, improve the flavour hierarchy of the fermented juice or enhance the conversion of natural active components. Furthermore, studies on optimising the processing technology for composite fruit juice fermentation remain limited.

Therefore, this study aimed to develop a peach–apricot juice through mixed-strain fermentation. To achieve this, 11 food-grade microbial strains with GRAS status were selected. These strains were originally isolated from fermented dairy products or fermented fruits and vegetables, characterised as facultative anaerobes, and this makes them highly adapted to fruit-based fermentation environments. Notably, the selected strains encompass multiple genera, including *Lactobacillus*, *Bifidobacterium*, and *Pediococcus*, with distinct functional traits within each genus. For example, *Lactiplantibacillus plantarum* subsp. *plantarum* has a strong acid-producing capacity, while *Levilactobacillus brevis* excels at enhancing flavour profiles. This functional diversity provides a broad genetic and phenotypic basis for subsequent single-strain screening. Four bacterial strains suitable for fermenting flat peaches and four strains suitable for fermenting Kuqa white apricots were selected based on their acid-, superoxide dismutase (SOD)- and flavour compound–producing capacities. These strains were used as starter cultures for developing fermented peach–apricot mixed juice (PAMJ). Subsequently, uniform design experiments were performed to determine the inoculation ratios of multi-strain mixtures for each fruit juice. Based on preliminary results, the flavour profiles of FPJ, FAJ, fermented peach–apricot blended juice (PABJ, a mixture of FPJ and FAJ) and PAMJ (fermented after mixing peach and apricot juices) were compared. PAMJ was identified as the optimal fermentation matrix. Subsequently, response surface methodology (RSM) was used to optimise fermentation parameters, with SOD activity, number of volatile compounds (NVC), an electronic tongue (ELT) and an electronic nose (ELN) serving as indicators. The optimal fermentation process for PAMJ was determined through quality comparison analysis. The storage stability of PAMJ at different temperatures was examined using pH, TSS, SOD, NVC, sensory scores and colour difference indices. Finally, a model to forecast PAMJ’s shelf life was established using the first-order kinetic and Arrhenius models. This study indicated that multi-strain fermentation prolonged the shelf life and enhanced the flavour of PAMJ, providing an innovative solution for speciality fruit processing.

## 2. Materials and Methods

### 2.1. Materials

Kuqa white apricots and Xinjiang flat peaches were purchased from local certified agricultural suppliers in Yining, Xinjiang. The fruits were hand-harvested at maturity and promptly transported to the laboratory under refrigerated conditions. Upon arrival, they were stored in cold storage at 4 °C before processing. Both varieties were botanically identified by Professor Wang Liang from the College of Life Science and Technology. *Lactiplantibacillus plantarum* subsp. *plantarum* (*L. plantarum*) CICC 25125, *Levilactobacillus brevis* (*L. brevis*) CICC 20014, *Limosilactobacillus reuteri* (*L. reuteri*) CICC 6123, *Lactobacillus acidophilus* (*L. acidophilus*) CICC 6086, *Lacticaseibacillus paracasei* subsp. *Tolerans* (*L. paracasei*) CICC 6109, *Limosilactobacillus fermentum* (*L. fermentum*) CICC 25124, *Lacticaseibacillus casei* (*L. casei*) CICC 6114, *Lactobacillus delbrueckii* subsp. *bulgaricus* (*L. delbrueckii*) CICC 6098 and *Pediococcus pentosaceus* (*P. pentosaceus*) CICC 21865 were obtained from the China Center of Industrial Culture Collection (Beijing, China). *Bifidobacterium thermophilum* (*B*. *thermophilum*) BIO-04950 and *Pediococcus acidilactici* (*P. acidilactici*) BIO-097553 were obtained from the Biobw Biotechnology Co., Ltd. (Beijing, China). Pectinase, cellulase and hemicellulase were purchased from Yuanye Biotechnology Co., Ltd. (Shanghai, China). A SOD activity assay kit was purchased from Nanjing Jiancheng Bioengineering Institute (Nanjing, China). Analytical-grade glacial acetic acid was obtained from Sheng’ao Chemical Reagent Co., Ltd. (Tianjin, China). deMan, Rogosa and Sharpe (MRS) broth was obtained from Aoboxing Biotechnology Co., Ltd. (Beijing, China).

### 2.2. Inoculum Preparation

1 mL of frozen bacterial suspension was aspirated and inoculated into 10 mL of sterile MRS medium, followed by incubation at 37 °C under dark and static conditions for 24–48 h. Subsequently, the activated bacterial suspension was inoculated into a fresh 100 mL of sterile MRS medium, which was then placed in a 37 °C incubator for 24–48 h. This step was repeated twice to ensure optimal growth.

### 2.3. Bacterial Density Counting

The activated bacterial suspension was diluted using a 10-fold serial dilution method. Specifically, 1 mL of the bacterial suspension was added to 9 mL of sterile water and vortexed thoroughly to prepare a 10^−1^ dilution. Then, 1 mL of this 10^−1^ dilution was transferred into another 9 mL of sterile water and mixed again to achieve a final dilution factor of 10^−2^ (100-fold). All apparatus and reagents were sterilised to mitigate the risk of contamination. Subsequently, 10 μL of the dilution was transferred to the edge of a sterilised hemocytometer’s counting chamber, allowing it to fill via capillary action without entrapping air bubbles. The hemocytometer was positioned beneath a microscope, and with appropriate magnification, bacterial cells within designated grids were counted.

For determining bacterial density in juice during fermentation, the bacteria in the fermented juice were counted hourly. The juice sample was first diluted 100-fold to ensure precise enumeration. Following the same loading procedure as described above, the diluted sample was introduced into the hemocytometer. Under appropriate magnification, bacterial cells were counted within grids to calculate the density.

### 2.4. Fermented Juice Preparation

FPJ: Flat peaches were cleaned and pulped. The pulp was subjected to enzymatic hydrolysis using 0.4% (*w*/*v*) pectinase, 0.2% (*w*/*v*) cellulase and 0.2% (*w*/*v*) hemicellulase at 55 °C for 3 h. After hydrolysis, sucrose was added to adjust the initial TSS content to 12%. Subsequently, the pulp was transferred into sterile screw-cap bottles, sterilised at 85 °C for 30 min, and then cooled. Afterwards, it was inoculated with the activated strains and fermented under static conditions in the dark at 37 °C for 36 h. The bottles remained sealed throughout the entire fermentation process.

FAJ: Apricots were processed using the same method as that described for flat peaches. After enzymatic hydrolysis, sucrose was added to adjust the initial TSS content to 18%. The pulp was transferred into sterile screw-cap bottles and sterilised at 85 °C for 30 min, cooled, inoculated with activated strains and fermented under static conditions in the dark at 37 °C for 36 h.

PAMJ: The pulps of flat peaches and apricots were mixed in a 2.5:1 ratio, followed by enzymatic hydrolysis. Subsequently, sucrose was added to adjust the initial TSS content to 16%, and the mixed pulp was transferred into sterile screw-cap bottles and sterilised at 85 °C for 30 min, cooled, inoculated with activated strains and fermented at 37 °C in the dark under static conditions for 36 h.

PABJ: FPJ and FAJ were mixed in a 2.5:1 ratio to prepare PABJ.

### 2.5. Selection of Dominant Strains and Determination of Their Proportions

Flat peach and white apricot pulps were individually inoculated with nine bacterial strains and incubated at 37 °C for 36 h. At the end of fermentation, the SOD activity, NVC, pH, TSS content and sensory scores of fermented products were determined to identify strains with excellent fermentation performance. In order to determine the optimal inoculum ratio of the four bacterial strains, a uniform design table featuring four factors and nine levels was employed (Table 1 and Table 2). The SPSS (version 26.0) software was used to derive a regression equation using SOD activity as the response variable. Calculations were performed using Excel.

The strains required for fermenting PAMJ (Table 3) were selected based on the strains identified from FPJ and FAJ. To obtain the optimal number of six bacteria in an inoculum, a uniform design table with 6 factors and 10 levels was used This step uses the same method for PAMJ as that applied to FPJ and FAJ.

### 2.6. Single-Factor Experiments

After fermentation strains and their proportions for FPJ, FAJ and PAMJ were selected, single-factor experiments were performed using fermentation time, fermentation temperature, inoculum amount and initial TSS content as variables and SOD activity and NVC as indicators to optimise the fermentation conditions. The specific experimental design is shown in Table 4.

### 2.7. Response Surface Optimisation

To optimise the fermentation process of PAMJ, a Box–Behnken design (BBD) was used, with temperature, time, inoculum concentration and initial TSS content as independent variables and SOD activity and NVC as response values. The factors and levels of the BBD are presented in Table 5.

### 2.8. Determination of Physicochemical Indices

SOD activity was determined following the operational protocol provided with the SOD assay kit. Absorbance measurements for the samples were conducted at a wavelength of 550 nm. To evaluate the colour in fermented samples, a colorimeter (DS-700D, Hangzhou Spectrum Technology Co., Ltd., Hangzhou, China) was utilised following calibration. TSS content was measured using a refractometer (model TD-45, Zhejiang Top Cloud-Agri Technology Co., Ltd., Hangzhou, China): 1 mL of each sample was pipetted into the refractometer injector, and readings were recorded. The pH value was measured with a digital pH metre (PHS-3C, Shanghai Leici Instrument Co., Ltd., Shanghai, China) [13]. VOCs were identified and quantified via a gas chromatography–mass spectrometry (GC–MS) system (8890-7000D, Agilent Technology Co., Ltd., Beijing, China). The specific GC–MS parameters are shown in Table 6 [14].

An ELN (C-PEN3, Beijing Enovel Technology Development Co., Ltd., Beijing, China) was used to analyse the odour of fermented samples. Briefly, 25 mL of each sample was placed in a vacuum bottle and left undisturbed at 25 °C for 0.5 h before detection. The detection parameters were set out as follows: detection duration, 120 s; injection flow rate, 400 mL/min and cleaning time, 80 s [15]. All sample measurements were conducted in triplicate, and the data are presented as average values.

An ELT (402 B single bond C, Beijing Enovel Technology Development Co., Ltd., Beijing, China) was used to evaluate the flavour characteristics of fermented samples. Briefly, 75 mL of each sample was transferred into the sample reservoir of the testing instrument. Subsequently, the pre-activated sensors were sequentially immersed into the liquid matrix following the preprogrammed testing protocol to conduct the analysis. Each sample was tested three times, and the data are presented as average values.

### 2.9. Sensory Evaluation

Sensory evaluation was performed as described by Liu et al. [16]. The sensory scoring group consisted of 20 professionally trained tasters (equal number of men and women, aged 25–40 years). Training and assessment were conducted following guidelines established by the International Organization for Standardization to ensure the accuracy of the evaluation process. Detailed criteria for sensory evaluation are mentioned in Table 7.

### 2.10. PAMJ Storage Stability Analysis

Following the completion of PAMJ fermentation, the product is filtered, transferred into brown glass bottles for sealing, and then subjected to pasteurisation at 85 °C for 30 min. The pasteurised PAMJ is then stored at various temperatures (4 °C, 25 °C, and 37 °C) to assess its storage stability. The storage time was 45 days. During the storage period, samples were taken every 5 days to measure SOD, NVC, pH, TSS.

### 2.11. Statistical Analysis

Experimental data obtained from three parallel experiments were expressed as the mean ± standard deviation or average values. Significance analysis of parallel data and between-group data was analysed using SPSS 26. All figures were drawn using GraphPad Prism v9.5. RSM data optimisation was performed using the Design-Expert 13 software. The statistical significance of the items in the regression equation was tested. Significant terms in the model were found by performing an analysis of variance (ANOVA) for each response.

## 3. Results and Discussion

### 3.1. Determination of Fermentation Strains

LAB exhibit significant specificity in fruit and vegetable fermentation. Studies have shown that different LAB strains exhibit distinct fermentation characteristics when cultured in the same fermentation matrix [17]. Therefore, we used various LAB strains to ferment peach juice and apricot juice individually. Dominant strains were selected from nine LAB strains using NVC, SOD activity and sensory scores as evaluation indices. Subsequent analysis focused on mixed-strain fermentation conditions to develop products with excellent flavour and antioxidant properties.

As shown in Table 8, fermentation with LAB strains significantly increased the SOD activity of FPJ. SOD acts as a pivotal antioxidant, exerting a significant role in safeguarding cells against oxidative stress [18]. The SOD activity of flat peach juice fermented with *L. reuteri*, *L. brevis*, *L. fermentum* and *L. paracasei* increased by 40.72, 28.87, 32.32 and 39.85 U/mL compared to that before fermentation, respectively. This increase may be related to the activation of endogenous enzymes and release of phenolic compounds, both of which increase the content of bioactive compounds, during fermentation [19]. After reaching a peak, SOD activity decreased with an increase in fermentation time. This pattern may be attributed to the stagnation of microbial growth in the later stages of fermentation, resulting in a lower SOD synthesis rate than its degradation rate. In addition, the reduced levels of antioxidants may further accelerate SOD degradation. After fermentation with *L. reuteri*, *L. brevis*, *L. fermentum* and *L. paracasei*, the NVC in flat peach juice increased by 9, 9, 8 and 7 compared to that in unfermented juice, respectively. Flat peaches are rich in precursor components such as carbohydrates, organic acids and vitamins, which provide nutritional support for LAB metabolism [20]. Differences in the increased NVC among peach juices fermented with different strains are associated with the specificity of their metabolic characteristics. Sensory evaluation directly indicates the quality of fermented juice, which is affected by strain type, appearance, aroma and taste. Flat peach juice fermented with *L. reuteri*, *L. brevis*, *L. fermentum* and *L. paracasei* achieved the highest sensory scores of 85.46, 85.03, 88.88 and 85.46, respectively. This difference may result from variations in the organic acid and VOC profiles produced by different strains during fermentation. Based on the comprehensive analysis of SOD activity, NVC and sensory scores, *L. reuteri*, *L. brevis*, *L. fermentum* and *L. paracasei* were selected as dominant strains for preparing FPJ.

Fermentation strains for FAJ were identified using the same method as that described in the case of FPJ. Results of single-strain fermentation of apricot juice are shown in Table 9. *L. plantarum*, *L. acidophilus*, *L. fermentum* and *L. brevis* significantly increased the SOD activity of apricot juice by 39.93, 38.27, 45.21 and 41.28 U/mL compared to that in unfermented juice, respectively. After fermentation with *L. plantarum*, *L. acidophilus, L. reuteri*, *L. fermentum* and *L. brevis*, the NVC in apricot juice increased by 6, 9, 6, 8 and 7, respectively. Apricot juice fermented with *L. plantarum*, *L. acidophilus*, *P. pentosaceus*, *L. fermentum* and *L. brevis* achieved the highest sensory scores of 85.43, 84.46, 85.26, 88.80 and 84.89, respectively. Based on the comprehensive assessment of SOD activity, NVC and sensory scores, *L. plantarum*, *L. acidophilus*, *L. fermentum* and *L. brevis* were selected as optimal strains for preparing FAJ. In addition, *L. fermentum*, *L. brevis*, *L. reuteri*, *L. paracasei*, *L. plantarum* and *L. acidophilus* were selected as optimal strains for preparing PAMJ.

### 3.2. Determination of the Proportions of Fermentation Strains

Studies have shown that the SOD activity of mixed-strain fermentation systems is higher than that of single-strain fermentation systems [21]. Consequently, mixed-strain fermentation technology was used in this study, and—a uniform design approach was adopted to determine the optimal ratio of fermentation strains. Detailed results of the uniform design experiments for FPJ, FAJ and PAMJ are presented in Table 1, Table 2 and Table 3, respectively.

(1) $$Y_{1}=111.91-0.0835\;X_{2}X_{3}+0.3898{X_{2}X}_{4}+0.0173X_{1}^{2}$$

In this regression equation, the R^2^ value was 0.9964, with a significance level of *p* < 0.01, indicating a good fit of the equation. This model accurately predicted the optimal fermentation conditions. The optimal inoculum proportions for FPJ were as follows: *L. reuteri*, 31.8%; *L. brevis*, 23.9%; *L. fermentum*, 17% and *L. paracasei*, 27.3%. Under these conditions, the predicted and experimentally measured SOD activities were 190 and 180 U/mL, respectively.

(2) $$Y_{2}=320-5.12X_{2}+0.018X_{1}X_{3}+1.632X_{2}X_{4}-0.275X_{2}^{2}-1.075X_{4}$$

For FAJ, the regression Equation (2) showed an R^2^ value of 0.9941 (*p* < 0.01), indicating a high goodness of fit. This model predicted the optimal inoculum proportions as follows: *L. brevis*, 25%; *L. fermentum*, 21.8%; *L. plantarum*, 37.5% and *L. acidophilus*, 15.7%. Under these conditions, the predicted and experimentally measured SOD activities were 378 and 368 U/mL, respectively.

(3) $$\begin{array}{cc} Y_{3}=262.19 & -\;17.712X_{1}+60.469X_{2}+0.5213X_{1}X_{6}-0.42804X_{2}X_{3}\\ & +\;0.02510X_{2}X_{4}-2.1038X_{2}X_{6}+0.60329X_{2}^{2}+0.062329X_{3}^{2} \end{array}$$

For PAMJ, the regression Equation (3) showed a high goodness of fit with an R^2^ value of 0.9941 (*p* < 0.01). The optimal inoculum proportions predicted by the model were as follows: *L. fermentum*, 15%; *L. acidophilus*, 10%; *L. brevis*, 34%.; *L. paracasei*, 13%; *L. plantarum*, 13% and *L. reuteri*, 15% Under these conditions, the predicted and experimentally measured SOD activities were 270 and 260 U/mL, respectively.

### 3.3. Effects of Different Fermentation Conditions on Juice Quality

**Figure 1:**
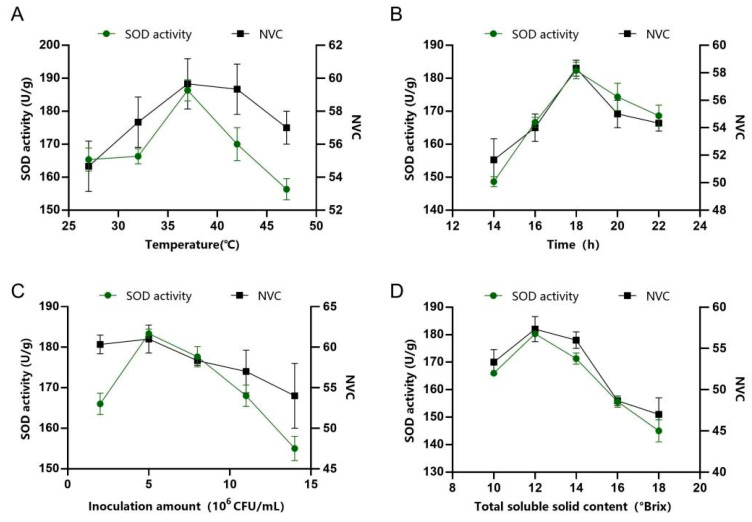
Results of single-factor experiments for FPJ. (**A**) Fermentation temperature, (**B**) fermentation time, (**C**) inoculum amount and (**D**) initial TSS content.

In the optimisation design of PAMJ fermentation conditions, the core optimisation parameters were selected as fermentation temperature, fermentation time, inoculation concentration and initial TSS based on the comprehensive consideration of microbial fermentation efficiency, product quality and production cost. During juice fermentation, temperature exerts a notable influence on the fermentation process and can easily affect the quality of the fermented product. Temperature is the primary environmental factor affecting bacterial growth and metabolism, which directly affects the metabolic ability of microorganisms. Excessively high temperatures can lead to the loss of volatile aroma compounds in the fermented product, whereas excessively low temperatures may result in incomplete fermentation [22]. As shown in Figure 1A, the highest SOD activity of 186 U/mL was achieved at the fermentation temperature of 37 °C. SOD activity decreased as the temperature was increased above 37 °C. Similarly, NVC achieved its maximum value at 37 °C. At fermentation temperatures exceeding 37 °C, the decreases in SOD activity and NVC may be related to the adverse effects of excessive temperature. [20]. For this reason, 37 °C was identified as the optimal fermentation temperature. Fermentation time directly determines the accumulation of metabolites and production efficiency. In cases where the fermentation duration was <14 h, LAB fail to grow and reproduce sufficiently, leading to inadequate accumulation of metabolites. This phenomenon resulted in a significant decrease in SOD activity and NVC. Similarly, when the fermentation time was excessively long, SOD activity and NVC showed decreasing trends. This phenomenon may be attributed to nutrient depletion in the substrate, reduced microbial density and excessive accumulation of metabolic by-products in the later stages of fermentation. During the fermentation period of 14–18 h, SOD activity showed a rapid increase, peaking at 182 U/mL at 18 h. During the same period, NVC also peaked (58 types) at 18 h, after which it gradually decreased as the fermentation time was increased. Dynamic changes in antioxidant activity are closely related to the content of bioactive components, which significantly increases with an increase in fermentation time. These results indicate that optimising the fermentation time is key to enhancing the quality of fermented products. Therefore, in subsequent experiments, the optimal fermentation time for FPJ was 18 h. The inoculum concentration determines the initiation rate of fermentation. When the inoculum concentration is too low, bacterial strains colonise the juice slowly, requiring an extended period to enter the logarithmic growth phase. During this lag, the product becomes susceptible to contamination by foreign bacteria, which may lead to spoilage. As illustrated in Figure 1C, at an inoculum concentration was 2 × 10^6^ CFU/mL, the bacterial population was relatively small, resulting in a slow metabolic rate. This decrease led to a reduction in SOD activity and the content of flavour compounds. At the inoculum concentration of 5 × 10^6^ CFU/mL, SOD activity reached 183 U/mL. However, when the inoculum concentration exceeded 1.1 × 10^7^ CFU/mL, excessive fermentation occurred, which reduced the stability of terpenoids and led to the loss of enzyme activity. Meanwhile, a high inoculum size also increases the overall strain cost. Therefore, the optimal inoculum concentration was determined to be 5 × 10^6^ CFU/mL. Initial TSS (primarily composed of carbohydrates such as sucrose, fructose, and glucose) serves mainly as a carbon source for microbial growth and metabolism. Both excessively high and low TSS levels can disrupt the flavour balance of the product by affecting carbon source availability and enzyme activity. As shown in Figure 1D, when the initial TSS content was adjusted to 12 °Brix, both SOD activity (180 U/mL) and NVC (51 types) were the highest. Therefore, 12 °Brix was selected as the optimal initial TSS content for fermentation. Finally, peach juice was fermented under the following conditions: fermentation duration of 18 h, temperature of 37 °C, inoculum concentration of 5 × 10^6^ CFU/mL and initial TSS content of 12 °Brix. Under these optimised conditions, the verified SOD activity of FPJ was 200 U/mL, and NVC was 61.

**Figure 2:**
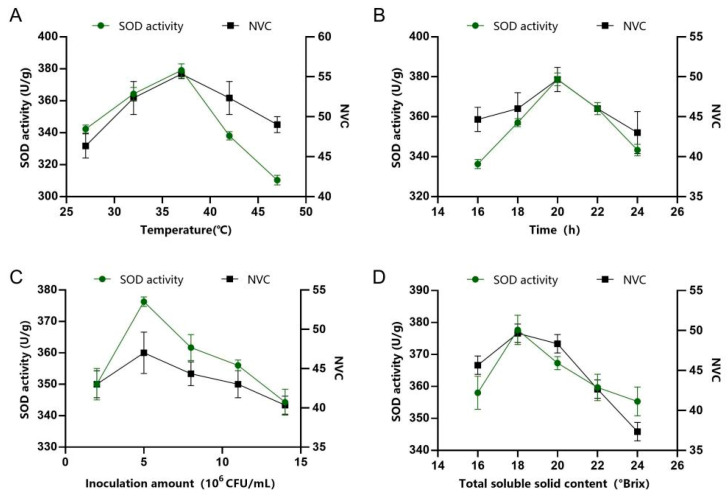
Results of single-factor experiments for FAJ. (**A**) Fermentation temperature, (**B**) fermentation time, (**C**) inoculum amount and (**D**) initial TSS content.

**Figure 3:**
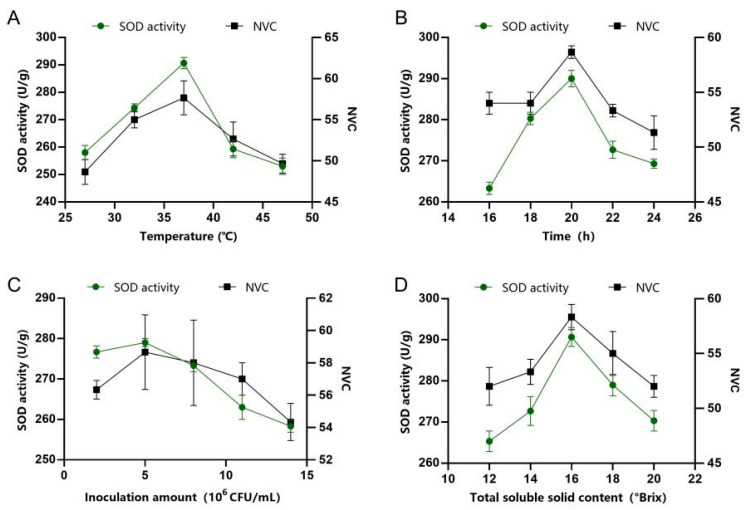
Results of single-factor experiments for PAMJ. (**A**) Fermentation temperature, (**B**) fermentation time, (**C**) inoculum amount and (**D**) initial TSS content.

The results of single-factor experiments for FAJ are shown in Figure 2. Apricot pulp underwent fermentation using the following parameters temperature of 37 °C, duration of 20 h, inoculum concentration of 5 × 10^6^ CFU/mL and initial TSS content of 18 °Brix. Under these conditions, the SOD activity of FAJ was 385 U/mL and NVC was 59. The results of single-factor experiments for PAMJ are shown in Figure 3. The mixed pulp of flat peaches and apricots was fermented under the following conditions: temperature of 37 °C, duration of 20 h, inoculum amount of 5 × 10^6^ CFU/mL and initial TSS content of 16 °Brix. Under these conditions, the SOD activity of PAMJ was 285 U/mL and NVC was 62. The prepared FPJ and FAJ were mixed in a ratio of 2.5:1 to obtain PABJ. PABJ had SOD activity of 250 U/mL and contained 60 species of VOCs.

### 3.4. Comparative Analysis of the Quality of Fermented Juices

#### 3.4.1. Sensory Evaluation Results

**Figure 4:**
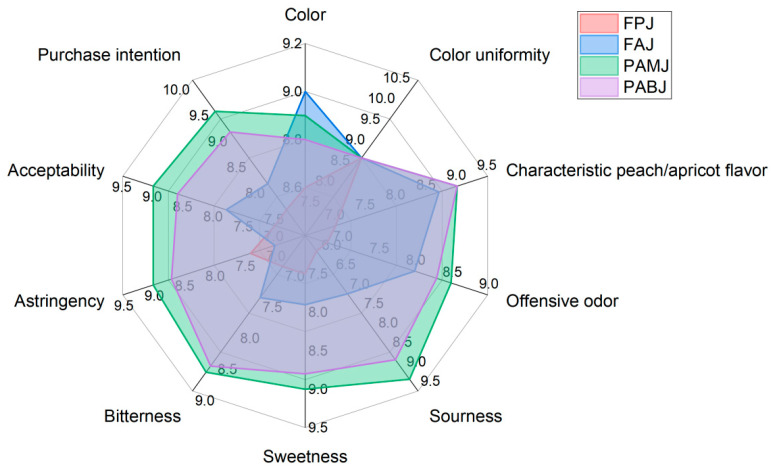
Radar chart of sensory scores for the four fermented juices.

As shown in Figure 4, all four fermented juices (PAMJ, FPJ, PABJ and FAJ) exhibited a transparent appearance with no impurities and good colour. Sensory evaluation indicated that PAMJ had the best peach and apricot flavours and the highest scores for sourness and sweetness. The sensory panel rated its sweet–sour balance as the most acceptable. FPJ developed a distinct off-flavour after sterilisation, likely attributed to heat-induced off-odours caused by the heat sensitivity of flat peaches. It received the lowest scores for both sourness and sweetness (7.7 points each), presumably because the high initial sweetness of the flat peach pulp was not ideally balanced by organic acids produced during fermentation [23]. FAJ had a distinct astringent taste. PABJ had relatively high scores for sourness (8.7 points) and sweetness (8.5 points); however, its bitter aftertaste significantly affected the overall mouthfeel. In terms of the overall sensory quality, the juices were ranked as follows: PAMJ (89.8 points) > PABJ (86.4 points) > FAJ (82.7 points) > FPJ (79.3 points). PAMJ was found to have the optimal overall sensory quality.

#### 3.4.2. ELT Analysis Results

**Figure 5:**
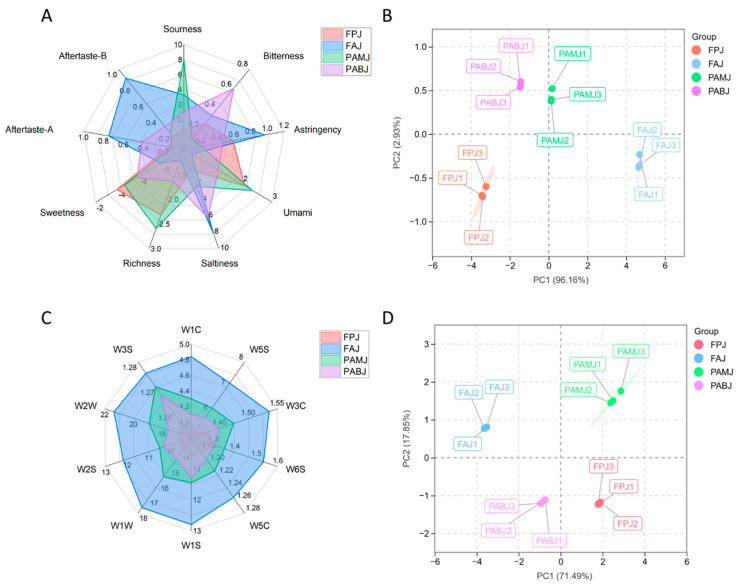
(**A**) Radar chart of ELT results for the four fermented juices, (**B**) PCA plot of ELT results for the four fermented juices, (**C**) radar chart of ELN results for the four fermented juices and (**D**) PCA plot of ELN results for the four fermented juices.

ELTs can simulate human taste perception through sensors. Figure 5A shows ELT results for the four fermented juices. Significant differences were found in the nine taste indices among all groups. In particular, FAJ exhibited the highest sourness response value of 3.32, whereas PAMJ exhibited a sourness response value of 1.54, which was significantly higher than that of PABJ and FAJ. In terms of sweetness, FPJ showed the highest response value of −3.93, whereas FAJ had the lowest response value of −7.92, with PAMJ showing an intermediate value. In terms of saltiness, FAJ had the highest response value of 8.18, whereas FPJ had the lowest response value of 2.33. The response values for bitterness and astringency showed minor differences among the four fermented juices. However, the human perception threshold for bitterness is low, leading to a strong perception of bitterness even with small values. ELT results indicated that PABJ had the highest bitterness response value of 0.61, whereas PAMJ had the lowest response value of 0.1. These results are consistent with those of sensory evaluation. FAJ had excessively high sourness and low sweetness, whereas FPJ had excessively high sweetness and low sourness. These attributes resulted in low overall acceptability for both juices. Although PABJ showed relatively good overall acceptability, its higher bitterness response value negatively affected the final mouthfeel. Principal component analysis (PCA) was performed on the ELT results. Results revealed differences among the four fermented juices, with the cumulative explanatory rate of principal components reaching 98.1% (Figure 5B). PAMJ had bitterness, richness and umami flavour as its main taste attributes, whereas FAJ was dominated by astringency, astringent aftertaste, bitter aftertaste and sourness. FAJ had the most prominent sourness, which may be attributed to the production of acidic substances such as lactic acid during fermentation [10]. PAMJ showed positive correlations with bitterness, richness and umami flavour but negative correlations with astringency and astringent aftertaste. Significant differences in flavour were observed among the four fermented juices, indicating that different processing methods exerted significant impacts on the flavour characteristics of the juices.

#### 3.4.3. ELN Analysis Results

ELNs can identify and distinguish different types of flavour compounds in a sample. Figure 5C shows ELN results for the four fermented juices. Both raw materials and the proportion of bacterial strains affected the flavour of fermented juices. Ten sensors exhibited differences in their response to FPJ, FAJ, PAMJ and PABJ. Among these sensors, W2W, W2S, W1W, W1S and W5S exhibited high response values, and differences among the four fermented juices were primarily reflected in the response intensities of these sensors. The response intensities of W2W, W2S, W1W, W1S and W5S sensors in PAMJ were 34.08%, 12.65%, 20.61%, 26.00% and 24.57% higher than those in FPJ, respectively. For PABJ, the response intensities of these sensors increased by 14.76%, 6.96%, 4.55%, 8.21% and 4.54%, respectively. For FAJ, the response intensities of these sensors increased by 35.93%, 21.09%, 24.58%, 28.01% and 21.36%, respectively. Other sensors, including W3S, W1C, W3C, W6S and W5C, showed very low response values, indicating extremely low contents of long-chain alkanes, benzenes, amines, hydrogen and alkanes in the four fermented juices.

PCA results are shown in Figure 5D. The first (PC1, 89.9%) and second (PC2, 7.6%) principal components had a cumulative contribution rate of 97.5%, indicating that this method effectively extracted the main features of the data with good reproducibility among samples. The four samples were distributed along the X-axis and Y-axis without any overlapping points, indicating significant and clearly distinguished differences among the aroma profiles of the four fermented juices.

#### 3.4.4. GC–MS Analysis Results

**Figure 6:**
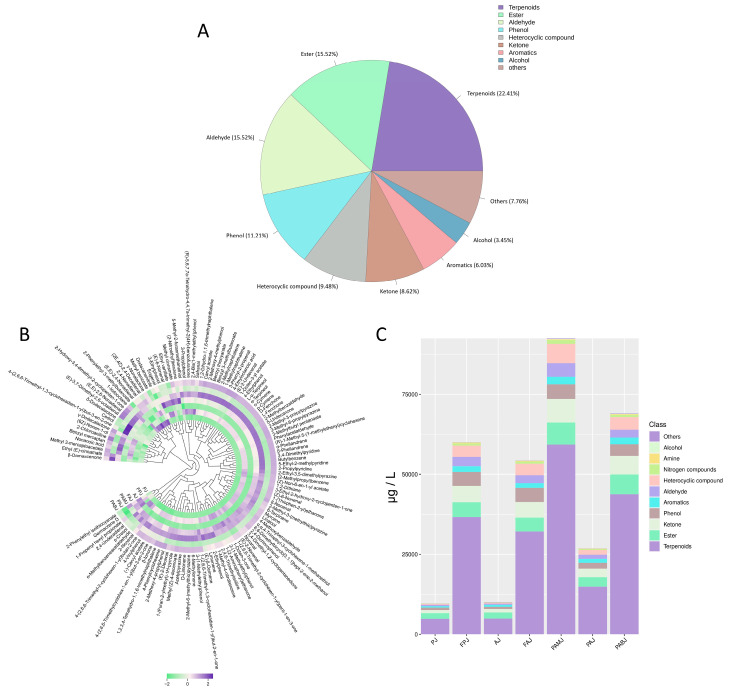
(**A**) VOCs component classification of all groups; (**B**) VOCs heat map, (**C**) Volcano plot of VOCs classification.

As shown in Figure 6, GC–MS analysis revealed a total of 116 VOCs across the four fermented juices. These VOCs included 26 terpenes, 18 aldehydes, 18 esters, 13 phenols, 11 heterocyclic compounds, 10 ketones, 7 aromatic hydrocarbons, 4 alcohols, 2 acids and 7 other compounds. These results are consistent with the ELN results. Among the VOCs, terpenes accounted for the highest relative concentration, followed by ketones and heterocyclic compounds. PAMJ exhibited the highest total VOC concentration of 92,462.48 μg/L, which was significantly higher than that of FAJ (5483.33 μg/L). Notably, the VOC concentrations of FPJ, FAJ and PAMJ significantly increased after fermentation, indicating that fermentation generated key flavour compounds.

Terpenoids significantly contribute to flavour owing to their low sensory threshold and unique floral and fruity notes [24]. Their content was particularly high in PAMJ. The concentrations of characteristic terpenoids such as δ-terpinene, α-terpineol, L-fenchone and linalool in PAMJ were 4.5, 5.6, 4.9 and 4.3 times higher than those in the unfermented sample, respectively. In addition, β-ocimene, D-limonene, myrcene, β-ionone, β-damascenone and other characteristic VOCs of flat peaches and white apricots collectively imparted PAMJ with rich peach and apricot flavours [25]. Consistent with these findings, a previous study showed that mixed fermentation with *Saccharomyces cerevisiae* and *L. plantarum* promoted the production of VOCs in citrus juices [26]. On the contrary, the concentrations of the abovementioned characteristic VOCs were generally lower in FPJ than in PAMJ, which may be attributed to the ability of mixed-strain fermentation to generate a greater diversity and quantity of VOCs [27].

Aldehydes, primarily derived from amino acid and fatty acid metabolism, are prone to transformation during fermentation [11]. Compared with the other three juices, PAMJ contained significantly higher levels of aldehydes such as 3-phenyl-2-propenal and α-methylphenylacetaldehyde. FPJ and FAJ accumulated relatively fewer aldehydes during fermentation, whereas PABJ showed an intermediate aldehyde content. Complex raw materials may provide more unsaturated fatty acid precursors, promoting the biosynthesis of aldehydes [28]. Esters are important aroma compounds in peach and apricot juices and are mainly produced through esterification reactions between alcohols and acids [29]. Both the diversity and content of esters in PAMJ were significantly higher than those in FAJ and FPJ. For example, compounds such as 1-octen-3-yl acetate, 3-methylbutyl valerate and methyl benzoate were highly concentrated in PAMJ. Furthermore, PAMJ contained significantly higher concentrations of key ketonic compounds, such as 3-ethyl-2-hydroxy-2-cyclopentenone, 3,4-dimethyl-1,2-cyclopentanedione and 1-(2,6,6-trimethyl-1,3-cyclohexadien-1-yl)-2-buten-1-one. These compounds have low sensory thresholds and substantially contribute to flavour.

Previous studies have used Lactobacillus plantarum to ferment peach pulp and white apricot pulp separately, and the results showed that this lactic acid bacterium could significantly enhance the production of aroma compounds in both pulps, with a particularly notable increase in alcohols and aldehydes [10,11]. A similar trend was observed in the present study. Current research mostly focuses on the fermentation process of single fruits, while systematic research on the mixed co-fermentation of two types of fruits remains relatively scarce. Furthermore, the vast majority of current fermentation studies still focus on a single fermentation method, and very few studies systematically compare the differences in the impacts of two processes (mixing raw materials before fermentation and mixing juices after fermentation) on the flavour of the final product. This study found that the co-fermentation of blended peach and apricot juice yields a more complex and desirable flavour profile compared to blending the two juices after their separate fermentation. This conclusion was validated through a comprehensive flavour assessment combining ELN, ELT and GC-MS analysis. Co-fermentation system produced a greater variety and abundance of volatile flavour compounds than either single-juice fermentations or post-fermentation blended juice, collectively creating a more complete flavour profile and enhancing the product’s overall sensory quality. Additionally, PAMJ exhibited a highly distinctive aroma profile. Its superior flavour characteristics originated from the synergistic contribution of characteristic terpenoids, aldehydes, esters, and ketones at elevated concentrations. Terpenoids provided natural floral and fruity notes, aldehydes contributed fresh nuances, esters delivered a soft creamy aroma, while ketones enhanced overall complexity. The well-balanced composition of these four compound classes constituted the core flavour advantage of PAMJ. Such outcomes align with prior studies, which note that mixed-strain fermentation can effectively boost flavour [30]. These findings strongly validated the scientific rationale and practical feasibility of the peach–apricot mixed co-fermentation strategy. Owing to these pronounced flavour advantages, PAMJ was selected as the target for subsequent optimisation using response surface methodology, establishing a solid foundation for further enhancement of the product’s functional properties.

#### 3.4.5. Optimal Fermentation Process of PAMJ Determined Through RSM

Preliminary experiments revealed that four factors (time, inoculum concentration, TSS, temperature) influenced both SOD activity and the NVC. A response surface experiment was designed based on the abovementioned results. Results of this experiment are shown in Table 8, and the quadratic polynomial regression equation is shown below.

(4) $$\begin{array}{cc} Y_{\mathrm{SOD}}=269.6 & -\;4.42X_{1}+2.17X_{2}-6.08X_{3}-4.33X_{4}-12X_{1}X_{2}+0.75X_{1}X_{3}\\ & -\;2X_{1}X_{4}-1.75X_{2}X_{3}-12.25X_{2}X_{4}+0.25X_{3}X_{4}\\ & -\;11.76{X_{1}}^{2}-21.13{X_{2}}^{2}-17.26{X_{3}}^{2}-18.88{X_{4}}^{2} \end{array}$$

(5) $$\begin{array}{cc} Y_{\mathrm{NVC}}=65.6 & -\;3.75X_{1}-0.67X_{2}+2.25X_{3}-4.17X_{4}-6.755X_{1}X_{2}+1.25X_{1}X_{3}+0.75X_{1}X_{4}\\ & +\;X_{2}X_{3}-8.25X_{2}X_{4}-0.50X_{3}X_{4}-5.01{X_{1}}^{2}-4.88{X_{2}}^{2}-8.76{X_{3}}^{2}-6.38{X_{4}}^{2} \end{array}$$

As shown in Table 10, the response surface regression models for both SOD activity and NVC were extremely significant with a high goodness of fit, indicating that these models can effectively predict the optimal fermentation conditions. For the SOD activity model, the significant terms included the linear terms A, C and D; the interaction terms AB, AD and BD and all quadratic terms. For the NVC model, the significant terms included the linear terms A, C and D; the interaction terms AB, AC and BD and all quadratic terms. All other terms in both models were non-significant.

**Figure 7:**
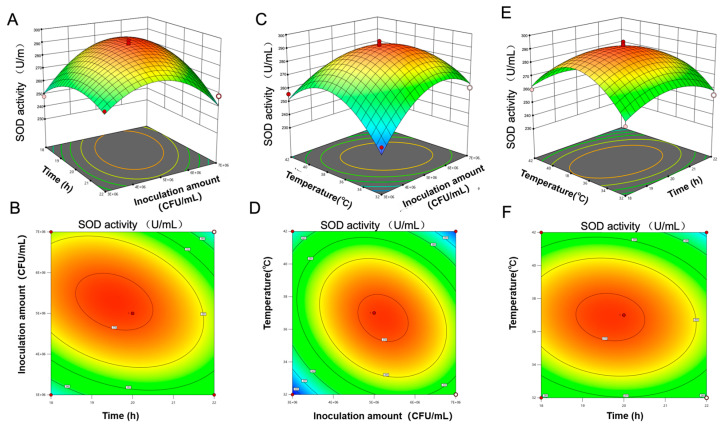
Effects of interactions between various factors on SOD activity. (**A**,**B**) Fermentation time and inoculum concentration, (**C**,**D**) fermentation temperature and inoculum concentration and (**E**,**F**) fermentation time and temperature.

**Figure 8:**
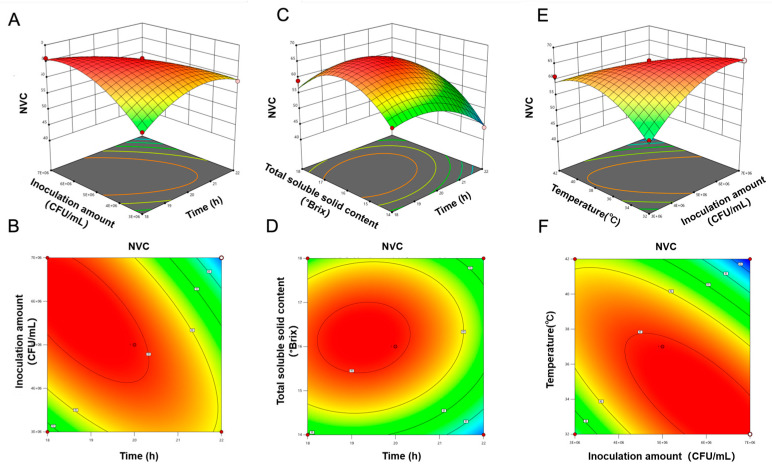
Effects of interactions between various factors on NVC. (**A**,**B**) Fermentation time and inoculum concentration, (**C**,**D**) fermentation time and initial TSS content and (**E**,**F**) fermentation temperature and inoculum concentration.

Three-dimensional response surface analysis was used to visualise the effects of fermentation conditions on SOD activity and NVC. As shown in Figure 7 and Figure 8, the slope of the response surface was positively correlated with the density of contour lines. The steeper the slope and the denser the contour lines, the more significant the effect. Elliptical contour lines indicated significant interactions between factors. SOD activity initially increased and subsequently decreased with an increase in the inoculum concentration at low temperatures. At high inoculum concentrations, SOD activity increased with an increase in temperature. The steep response surfaces and elliptical contour lines for temperature and inoculum concentration validated their significant synergistic effect. NVC increased with increasing inoculum concentrations at low temperatures. However, at high temperatures and high NVC values, SOD activity decreased significantly.

Response surface optimisation was performed using SOD activity and NVC as response values. The predicted optimal fermentation parameters are outlined as follows: fermentation duration of 19.54 h, temperature of 36.84 °C, inoculum concentration of 5.16 × 10^6^ CFU/mL and initial TSS content of 16.38 °Brix. Under such parameters, the predicted SOD activity reaches 290 U/mL, with the predicted NVC standing at 66. For ease of operation, the fermentation duration was adjusted to 20 h, temperature to 37 °C, TSS content to 16.5 °Brix and inoculum concentration to 5.2 × 10^6^ CFU/mL. Under these conditions, SOD activity was 295 U/mL and NVC was 6. These values were highly consistent with the predicted values.

### 3.5. Storage Stability and Shelf Life of PAMJ

#### 3.5.1. Different Storage Temperatures

**Figure 9:**
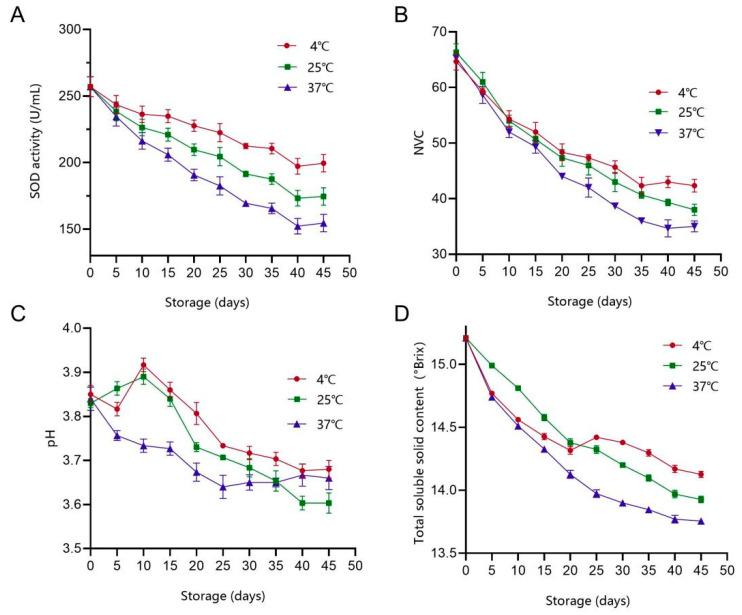
Changes in physicochemical indices at different storage temperatures. (**A**) SOD activity, (**B**) NVC, (**C**) pH and (**D**) TSS content.

**Figure 10:**
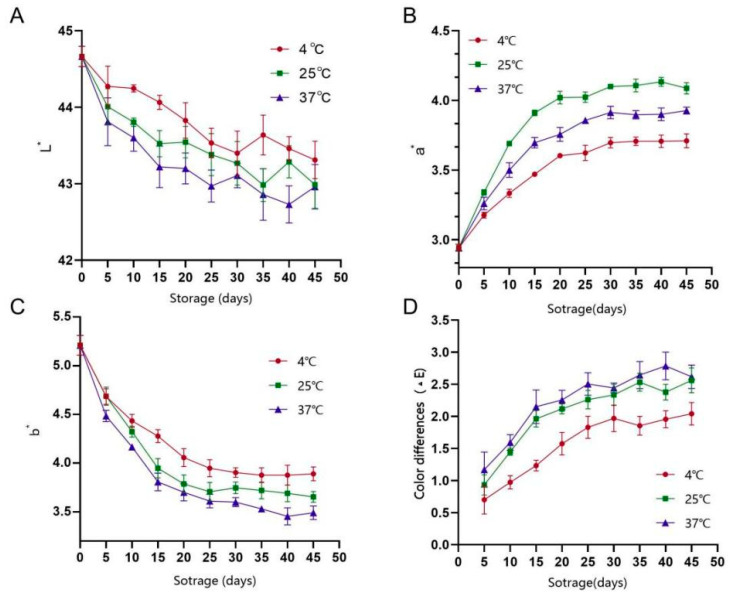
Changes in colour difference values at different storage times. (**A**) The lightness value, (**B**) red–green chromaticity, (**C**) yellow–blue chromaticity and (**D**) the colour difference value.

SOD is an important free radical scavenger in fermented fruit and vegetable juices [31]. SOD activity, as an important index of fermented plant beverages, directly affects their nutritional properties. This indicator has been used to screen suitable raw material matrices for LAB-driven fermentation [32]. As shown in Figure 9, SOD activity significantly decreased after 45 days of storage. The losses of SOD activity at 4 °C, 25 °C and 37 °C were 58, 82 and 102 U/mL, respectively. These findings are consistent with the changes in SOD activity observed during the storage of fermented kiwifruit juice [21]. Owing to its heat sensitivity, the stability of SOD is affected by environmental temperature, which may be an important reason for the differences in SOD activity at different temperatures [33]. Furthermore, we monitored changes in NVC at different storage temperatures and found that NVC showed a linear decreasing trend with increasing storage time (Figure 10B). At 4 °C, NVC decreased rapidly within the initial 20 days, followed by a slower rate of decrease. Over the 45-day storage, the total number of VOCs decreased by 23 species. At 25 °C and 37 °C, NVC decreased more significantly, with losses of 28 and 30 species, respectively. The degradation rates at these temperatures were 1.25 and 1.34 times higher than those at 4 °C, indicating a significant negative correlation between storage temperature and VOC stability. The effects of different storage temperatures on the pH of fermented juices were significantly different. At 4 °C, pH showed a decrease of 4.4% (from 3.85 to 3.68). At 25 °C, pH showed the largest decrease to 3.60. At 37 °C, pH slightly decreased from 3.85 to 3.66. Excessively high acidity may affect product acceptability and maintaining an appropriate sweet–sour balance is crucial for consumer acceptance. The pH decreases observed in this study are consistent with the findings reported by Lafarga et al. [33], who found that samples stored at 4 °C exhibited the best pH stability. This stability may be attributed to the inhibition of microbial metabolic activity at low temperatures [34]. As a key parameter for evaluating juice quality, TSS content not only affects product texture but also plays an important role in maintaining the stability of bioactive components. As shown in Figure 10D, TSS content in all temperature groups showed a decreasing trend during storage. The TSS contents of samples stored at 4 °C, 25 °C and 37 °C decreased from 15.21% to 14.12%, 13.92% and 13.75%, respectively. The degradation rate of TSS was most significant at 37 °C. Altogether, low-temperature storage was found to be more conducive to maintaining the stability of PAMJ.

#### 3.5.2. Analysis of Colour Change

As shown in Figure 10, storage temperature significantly affected the colour of PAMJ. The lightness (L*) value decreased with an increase in storage time. The largest decrease of 2% occurred at 37 °C, whereas the smallest decrease of 1% occurred at 4 °C, indicating that low temperatures delayed the loss of lightness. Red–green chromaticity (a*) increased in all groups, with the 25 °C group showing the most significant increase, followed by the 37 °C group. These findings suggested that higher temperatures led to more pronounced reddening. Yellow–blue chromaticity (b*) showed the most significant decrease of 45% in the 37 °C group, whereas the smallest decrease was observed in the 4 °C group. This decrease may be related to carotenoid degradation at high temperatures. To eliminate environmental errors and quantify colour changes, the colour difference value (ΔE) was used for evaluation (6). Results indicated that the ΔE value was significantly lower in the 4 °C group than in the other temperature groups. These findings indicate that an increase in storage temperature aggravates the browning of PAMJ, whereas low temperatures can effectively delay its colour change.

(6) $$∆E=\sqrt{({L_{2}{\;-\;L}_{1})}^{2}+(a_{2}{\;-\;a}_{1})+({b_{2}\;-\;b_{1})}^{2}}$$

#### 3.5.3. Changes in Total Colony Counts During Storage of PAMJ

Storage experiments on PAMJ were performed at different temperatures. The total colony count and the counts of yeasts and moulds were measured every 5 days, with the observation lasting 45 days. No microorganisms were detected in any group. Samples were retested on the 45th day of storage, and no microorganisms were detected. These findings indicated that canning sterilisation effectively killed microorganisms in PAMJ.

#### 3.5.4. Changes in the Sensory Quality of PAMJ

**Figure 11:**
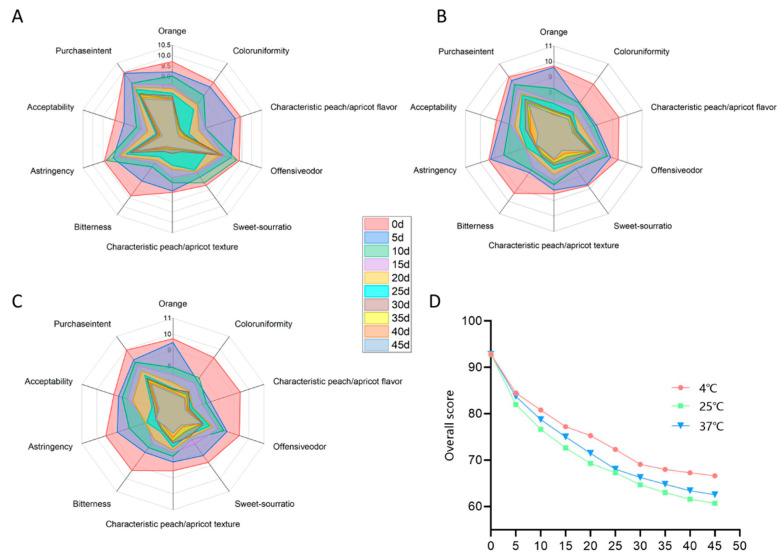
(**A**) Radar chart of sensory scores during 0–45 days of storage at 4 °C, (**B**) radar chart of sensory scores during 0–45 days of storage at 25 °C, (**C**) radar chart of sensory scores during 0–45 days of storage at 37 °C, (**D**) Overall sensory score during 0–45 days of storage at different temperatures.

As shown in Figure 11, the sensory scores of PAMJ at different storage temperatures dropped markedly as storage time progressed (*p* < 0.05). The initial sensory score of the sample was 92.8. After 45 days, the sensory scores of the 4 °C, 25 °C and 37 °C groups decreased to 75.4, 65.2 and 62.5, respectively. This pattern indicates that the higher the temperature, the more significant the deterioration in colour, flavour and texture. The samples stored at 4 °C had the best sensory quality. Although their sensory scores decreased, all samples retained acceptability during the 45-day storage. This phenomenon is mainly attributed to the inhibition of microorganisms caused by pasteurisation and the acidic environment. Changes in sensory scores were highly correlated with physicochemical properties during storage.

#### 3.5.5. Shelf-Life Prediction for PAMJ

A first-order kinetic model was used to fit and analyse the data of SOD activity and NVC under constant-temperature conditions. The first-order kinetic equation is shown below.

(7) $$A=A_{0}e^{\mathrm{kt}}$$

where *A*, *A*_0_, *k* and *t* denote the index value after storage for *t* days, the initial index value before storage, the reaction rate constant and the preservation time, respectively.

(8) $$\mathrm{In}\;k={\mathrm{In}\;k}_{0}-\frac{E_{a}}{8.314T}$$

where *k*_0_, *E_a_* and *T* denote the frequency factor, the activation energy (J/mol), and the absolute temperature, respectively.

(9) $$SL=\frac{In\;A\;-\;{In\;A}_{0}}{k_{0}{e^{-}}^{\frac{E_{a}}{RT}}}$$

(10) $$RT=\frac{X_{1}-X_{0}}{X_{0}}\;\times \;100\%$$

where X_0_ and X_1_ represent the measured value and predicted value, respectively.

**Figure 12:**
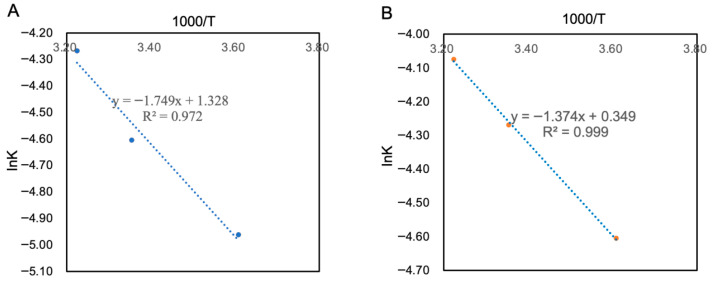
Arrhenius curve equations during storage. (**A**) SOD activity and (**B**) NVC.

The measured experimental data were fitted according to Equation (7), and the results are shown in Table 11. The *k* in the SOD activity model were 0.007, 0.010 and 0.014, whereas those in the NVC model were 0.010, 0.014 and 0.017. A scatter plot was drawn with In *k* as the *y*-axis and 1000/*T* as the *x*-axis, and the fitting results are shown in Figure 12. The slope and intercept of the linear equation in Figure 12 were substituted into Equation (8) to calculate *Ea* and *k*_0_. Then, the actual and predicted validity periods of PAMJ were calculated using Equation (9), whereas the relative error (*RT*) between these periods was calculated using Equation (10).

When SOD activity dropped to 50% of its initial value, the shelf life of PAMJ was 99 days at 4 °C, 63 days at 25 °C and 49 days at 37 °C. Similarly, when NVC activity dropped to 50% of its original level, the shelf life was 69 days at 4 °C, 48 days at 25 °C and 39 days at 37 °C. To validate the shelf-life prediction model, PAMJ was stored at 8 °C and 28 °C and the actual shelf life was measured (Table 12). The relative errors between model predictions and actual values remained <10%, indicating that the model efficiently predicted the shelf life of PAMJ. These findings provide a reliable basis for predicting changes in storage quality.

## 4. Conclusions

Probiotic fermentation technology effectively enhances the nutritional and sensory qualities of fruit juices. This study employed a systematic strategy involving single-strain screening, strain ratio optimisation, flavour comparison, and parameter refinement to significantly enhance both the functional and sensory qualities of PAMJ. Multidimensional flavour analysis clearly demonstrated the notable advantage of the strategy of blending before fermentation in promoting flavour synergy. Additionally, low-temperature storage effectively preserved the aroma, colour, and textural stability of the product, achieving a shelf life of up to three months. This research provides a systematic methodological reference and theoretical foundation for the application of mixed-culture fermentation technology in the deep processing of fruits and vegetables, thereby supporting the development of high-value-added fermented products and enhancing the added value of agricultural commodities.

## Figures and Tables

**Table 1:** Results of uniform design experiments for FPJ.

No.	*L. paracasei* X_1_ (%)	*L. brevis* X_2_ (%)	*L. reuteri* X_3_ (%)	*L. fermentum* X_4_ (%)	SOD Activity (U/mL)
1	16	15	34	15	162
2	17	18	31	14	168
3	18	21	28	13	175
4	19	14	35	12	143
5	20	17	32	11	145
6	21	20	29	10	150
7	22	13	36	9	126
8	23	16	33	8	129
9	24	19	30	7	125

**Table 2:** Results of uniform design experiments for FAJ.

No.	*L. brevis* X_1_ (%)	*L. fermentum* X_2_ (%)	*L. plantarum* X_3_ (%)	*L. acidophilus* X_4_ (%)	SOD Activity (U/mL)
1	16	15	34	15	315
2	17	18	31	14	349
3	18	21	28	13	365
4	19	14	35	12	328
5	20	17	32	11	340
6	21	20	29	10	335
7	22	13	36	9	324
8	23	16	33	8	321
9	24	19	30	7	302

**Table 3:** Results of uniform design experiments for PAMJ.

No.	*L. fermentum* X_1_ (%)	*L. acidophilus* X_2_ (%)	*L. reuteri* X_3_ (%)	*L. paracasei* X_4_ (%)	*L. plantarum* X_5_ (%)	*L. brevis* X_6_ (%)	SOD Activity (U/mL)
1	15	2	8	8	13	34	252
2	16	4	11	13	9	33	237
3	17	6	14	7	16	32	212
4	18	8	6	12	12	31	229
5	19	10	9	6	8	30	234
6	20	1	12	11	15	29	224
7	21	3	15	5	11	28	212
8	22	5	7	10	7	27	215
9	23	7	10	4	14	26	223
10	24	9	13	9	10	25	242

**Table 4:** Single-factor experiments.

	FPJ	FAJ	PAMJ
Fermentation temperature (°C)	27	27	27
	32	32	32
	37	37	37
	42	42	42
	47	47	47
Fermentation time (h)	16	16	16
	18	18	18
	20	20	20
	22	22	22
	24	24	24
Inoculum concentration (CFU/mL)	2 × 10^6^	2 × 10^6^	2 × 10^6^
	5 × 10^6^	5 × 10^6^	5 × 10^6^
	8 × 10^6^	8 × 10^6^	8 × 10^6^
	11 × 10^6^	11 × 10^6^	11 × 10^6^
	14 × 10^6^	14 × 10^6^	14 × 10^6^
Initial TSS content (°Brix)	10	16	12
	12	18	14
	14	20	16
	16	22	18
	18	24	20

**Table 5:** Response surface optimisation for PAMJ.

No.	Factors				Response Values	
	Time (h)	Inoculum Concentration (×10^6^ CFU/mL)	TSS (°Brix)	Temperature (°C)	SOD Activity (U/mL)	NVC (Types)
1	20	5	16	42	293	70
2	20	5	14	32	245	46
3	18	5	16	37	255	61
4	20	5	16	37	292	65
5	20	7	16	37	235	42
6	20	3	16	37	256	61
7	22	5	16	32	235	47
8	20	7	16	37	260	66
9	20	5	16	37	288	66
10	20	5	16	37	290	66
11	18	3	16	37	248	54
12	20	7	14	37	252	47
13	22	3	16	37	263	59
14	20	7	16	32	260	55
15	42	6	16	37	265	53
16	20	3	14	37	240	49
17	22	5	16	32	255	54
18	20	3	16	32	240	52
19	20	5	16	37	295	65
20	20	5	18	32	260	56
21	20	5	14	32	249	54
22	18	5	16	42	260	51
23	22	7	16	37	240	44
24	20	5	18	42	257	50
25	18	5	14	37	258	55
26	18	7	16	37	273	64
27	22	5	14	37	250	38
28	20	3	18	37	255	53
29	18	5	18	37	270	50

**Table 6:** GC–MS parameters.

Sample	FPJ/FAJ/PAMJ/PABJ
Capillary chromatographic column	DB-5MS (30 m × 0.25 mm × 0.25 µm)
Carrier gas	Helium
Carrier gas flow rate	1.2 mL/min
Injection mode	Splitless
Detector temperature	250 °C
Temperature programme	Initial column temperature of 40 °C held for 3.5 min
	Heated to 180 °C at 7 °C/min
	Heated to 280 °C at 25 °C/min, held for 5 min
Ion source temperature	150 °C
Interface temperature	280 °C
Electron energy	70 eV

**Table 7:** Sensory evaluation criteria.

Scoring Items	Scoring Criteria	Score
Appearance	Colour	0–10
	Colour uniformity	0–10
Aroma	Characteristic peach/apricot flavour	0–10
	Offensive odour	0–10
Taste	Sourness	0–10
	Sweetness	0–10
	Bitterness	0–10
	Astringency	0–10
Overall acceptability	Acceptability	0–10
	Purchase intention	0–10

**Table 8:** Physicochemical properties of flat peach juice fermented with a single bacterial strain.

No.	Strain	pH	Initial TSS Content (%)	SOD Activity (U/mL)	NVC (Types)	Sensory Score	Time to 10^8^ CFU/mL (h)
1	Unfermented	4.04 ± 0.01 ^b^	12.00 ± 0.01 ^b^	113.91 ± 0.10 ^a^	41 ± 0.75 ^cd^	80.00 ± 0.63 ^b^	/
2	*L. plantarum*	3.83 ± 0.02 ^a^	11.85 ± 0.02 ^c^	137.84 ± 8.64 ^bc^	46± 1.02 ^bc^	81.43 ± 1.34 ^d^	16.32 ± 0.32 ^b^
3	*L. casei*	3.87 ± 0.01 ^abc^	11.85 ± 0.16 ^de^	134.53 ± 8.23 ^b^	43 ± 0.55 ^cd^	82.13 ± 0.23 ^ab^	24.13 ± 0.32 ^ab^
4	*L. acidophilus*	3.87 ± 0.02 ^de^	11.8 ± 0.22 ^de^	126.18 ± 10.30 ^ab^	46 ± 1.17 ^bc^	84.46 ± 1.46 ^cd^	20.76 ± 0.24 ^ab^
5	*L. reuteri*	3.83 ± 0.02 ^g^	11.5 ± 0.11 ^de^	154.63 ± 7.45 ^bc^	50 ± 1.03 ^f^	85.46 ± 0.24 ^a^	19.96 ± 0.34 ^cd^
6	*P. pentosaceus*	3.89 ± 0.03 ^fg^	11.6 ± 0.23 ^de^	132.34 ± 4.62 ^e^	45 c ± 1.39 ^ef^	84.26 ± 1.45 ^a^	19.67 ± 0.43 ^a^
7	*L. brevis*	3.82 ± 0.03 ^c^	11.5 ± 0.08 ^bcd^	142.78 ± 2.24 ^d^	50 c ± 1.67 ^ab^	85.03 ± 1.34 ^ab^	17.83 ± 0.23 ^ab^
8	*L. fermentum*	3.82 ± 0.02 ^cd^	11.5 ± 0.06 ^a^	146.12 ± 13.31 ^a^	49 ± 1.42 ^de^	88.80 ± 0.52 ^cd^	18.70 ± 0.34 ^ab^
9	*P. acidilactici*	3.84 ± 0.03 ^d^	11.8 ± 0.07 ^b^	129.19 ± 5.34 ^ab^	46 ± 0.08 ^e^	84.89 ± 1.42 ^cd^	19.77 ± 0.62 ^bc^
10	*L. paracasei*	3.82 ± 0.02 ^a^	11.6 ± 0.11 ^abc^	153.76 ± 4.45 ^cd^	48 ± 1.41 ^de^	85.46 ± 0.34 ^abc^	18.85 ± 0.41 ^a^

Different lowercase letters in the upper right corner of the values in the same column represent significant differ-ences between the values (*p* < 0.05).

**Table 9:** Physicochemical properties of apricot juice fermented with a single bacterial strain.

No	Strain	pH	Initial TSS Content (%)	SOD Activity (U/mL)	NVC (Types)	Sensory Score	Time to 10^8^ CFU/mL (h)
1	Unfermented	3.62 ± 0.00 ^bc^	16.6 ± 0.08 ^ab^	287.91 ± 0.00 ^i^	43 ± 0.75 ^ef^	80.00 ± 0.82 ^e^	/
2	*L. plantarum*	3.46 ± 0.00 ^fg^	16.1 ± 0.14 ^de^	327.84 ± 8.98 ^ef^	49 ± 0.0.75 ^c^	85.43 ± 1.50 ^e^	20.32 ± 0.45 ^ab^
3	*L. casei*	3.48 ± 0.01 ^ab^	16.1 ± 0.26 ^de^	324.53 ± 8.16 ^de^	45 ± 1.02 ^a^	82.13 ± 0.30 ^a^	19.13 ± 0.19 ^ef^
4	*L. acidophilus*	3.47 ± 0.00 ^defg^	16.1 ± 0.08 ^e^	326.18 ± 10.90 ^gh^	52 ± 0.55 ^cd^	84.46 ± 1.48 ^ef^	18.76 ± 0.42 ^f^
5	*L. reuteri*	3.48 ± 0.01 ^g^	16.5 ± 0.12 ^de^	314.63 ± 7.59 ^ef^	49 ± 1.42 ^ef^	82.46 ± 0.53 ^fg^	20.96 ± 0.85 ^ab^
6	*P. pentosaceus*	3.48 ± 0.01 ^fg^	16.3 ± 0.00 ^cd^	302.34± 4.62 ^i^	45 ± 1.03^e^	85.26 ± 1.03 ^g^	19.67 ± 0.42 ^def^
7	*B. thermophilum*	3.48 ± 0.01 ^cde^	16.5± 0.19 ^bc^	322.78 ± 2.56 ^fg^	48 ± 1.72 ^bc^	81.03 ± 1.18 ^de^	20.83 ± 0.51 ^def^
8	*L. fermentum*	3.47 ± 0.02 ^cd^	16.2 ± 0.17 ^de^	333.12 ± 13.31 ^e^	51 ± 1.39 ^ab^	88.80 ± 0.64 ^c^	24.70 ± 0.33 ^cd^
9	*L. brevis*	3.47 ± 0.01 ^def^	16.1 ± 0.14 ^ab^	329.19 ± 5.65 ^h^	50 ± 1.67 ^ab^	84.89 ± 1.42 ^fg^	19.77 ± 0.16 ^a^
10	*L. delbrueckii*	3.55 ± 0.02 ^def^	16.3 ± 0.08 ^a^	313.76 ± 4.56 ^cd^	46 ± 1.51 ^cd^	82.46 ± 0.87 ^fg^	20.85 ± 0.43 ^de^

Different lowercase letters in the upper right corner of the values in the same column represent significant differences between the values (*p* < 0.05).

**Table 10:** ANOVA for the SOD activity and NVC models in PAMJ.

Source of Variation	SOD Activity (U/mL)		NVC (Compounds)	
	F Value	*p* Value	F Value	*p* Value
Regression model	83.15	<0.0001	48.55	<0.0001
(A) Time	25.55	0.0002	49.51	<0.0001
(B) Inoculum concentration	3.91	0.068	3.09	0.1004
(C) TSS	64.35	<0.0001	24.26	0.0002
(D) Temperature	5.33	0.0368	65.48	<0.0001
AB	83.46	<0.0001	58.02	<0.0001
AC	0.326	0.5771	37.14	<0.0001
AD	8.15	0.0127	0.8355	0.3761
BC	1.78	0.204	1.49	0.2431
BD	60.9	<0.0001	101.1	<0.0001
CD	0.0362	0.8518	0.3714	0.552
A^2^	193.32	<0.0001	117.75	<0.0001
B^2^	448.39	<0.0001	60.02	<0.0001
C^2^	270.57	<0.0001	240.48	<0.0001
D^2^	474.42	<0.0001	79.41	<0.0001
Lack of fit	0.9235	0.5846	0.4767	0.844
R^2^	0.9631	-	0.9738	-
R^2^_Adj_	0.9263	-	0.9596	-

**Table 11:** Regression equations and parameters for changes in indices over time at different storage temperatures.

Detection Index	Storage Temperature/K	Regression Equation	Reaction Rate Constant (k)	Regression Coefficient (R^2^)
SOD activity	277.15 (4 °C)	y = 265.01e^−0.007^	−0.007	0.960
	298.15 (25 °C)	y = 265.01e^−0.010^	−0.010	0.963
	310.15 (37 °C)	y = 265.01e^−0.014^	−0.014	0.951
NVC	277.15 (4 °C)	y = 65e^−0.010^	−0.010	0.958
	298.15 (25 °C)	y = 65e^−0.014^	−0.014	0.947
	310.15 (37 °C)	y = 65e^−0.017^	−0.017	0.951

**Table 12:** Comparison of the predicted and actual shelf life of PAMJ.

Storage Temperature (°C)	SOD Activity (U/mL)			NVC (Types)		
	Predicted Value	Actual Value	Relative Error	Predicted Value	Actual Value	Relative Error
8	90	96	6.25%	64	69	7.25%
28	60	65	7.69%	46	50	8.00%

## Data Availability

The original contributions presented in the study are included in the article; further inquiries can be directed to the corresponding author(s).

## Abbreviations

FPJ	fermented peach juice
FAJ	fermented apricot juice
PAMJ	fermented peach–apricot mixed juice
PABJ	fermented peach–apricot blended juice
SOD	superoxide dismutase
NVC	number of volatile compounds
TSS	total soluble solid
VOCs	volatile organic compounds
LAB	lactic acid bacteria
RSM	response surface methodology
ELT	electronic tongue
ELN	electronic nose
MRS	deMan, Rogosa and Sharpe
BBD	Box–Behnken design
GC–MS	gas chromatography–mass spectrometry
PCA	principal component analysis

## References

[1] Alajil O., Sagar V.R., Kaur C., Rudra S.G., Sharma R.R., Kaushik R., Verma M.K., Tomar M., Kumar M., Mekhemar M. (2021). Nutritional and phytochemical traits of apricots (*Prunus armeniaca* L.) for application in nutraceutical and health industry. Foods.

[2] Tan F., Wang P., Zhan P., Tian H. (2022). Characterization of key aroma compounds in flat peach juice based on gas chromatography-mass spectrometry-olfactometry (GC-MS-O), odor activity value (OAV), aroma recombination, and omission experiments. Food Chem..

[3] Raji R., Jannatizadeh A., Fattahi R., Esfahlani M.A. (2014). Investigation of variability of apricot (*Prunus armeniaca* L.) using morphological traits and microsatellite markers. Sci. Hortic..

[4] Wang C., Zhu X., Shao S., Huang M., Gou N., Zhang Y., Chen C., Bai H., Qu J., Huang Z. (2023). Metabolomic and transcriptomic analyses reveal novel mechanisms underlying the long-storage trait of apricot (*Prunus armeniaca* L.). Sci. Hortic..

[5] Li Y., Zhao Y., Zhang Z., He H., Shi L., Zhu X., Cui K. (2022). Near-freezing temperature storage improves shelf-life and suppresses chilling injury in postharvest apricot fruit (*Prunus armeniaca* L.) by regulating cell wall metabolism. Food Chem..

[6] Muhialdin B.J., Kadum H., Zarei M., Meor Hussin A.S. (2020). Effects of metabolite changes during lacto-fermentation on the biological activity and consumer acceptability for dragon fruit juice. LWT.

[7] Chen C., Lu Y., Yu H., Chen Z., Tian H. (2019). Influence of 4 lactic acid bacteria on the flavor profile of fermented apple juice. Food Biosci..

[8] Ricci A., Marrella M., Hadj Saadoun J., Bernini V., Godani F., Dameno F., Neviani E., Lazzi C. (2020). Development of lactic acid-fermented tomato products. Microorganisms.

[9] Wang L., Sun X., Li F., Yu D., Liu X., Huang W., Zhan J. (2015). Dynamic changes in phenolic compounds, colour and antioxidant activity of mulberry wine during alcoholic fermentation. J. Funct. Foods.

[10] Sun J., Zhao C., Pu X., Li T., Shi X., Wang B., Cheng W. (2022). Flavor and functional analysis of *Lactobacillus plantarum* fermented apricot juice. Fermentation.

[11] Yang W., Liu J., Zhang Q., Liu H., Lv Z., Zhang C., Jiao Z. (2022). Changes in nutritional composition, volatile organic compounds and antioxidant activity of peach pulp fermented by *Lactobacillus*. Food Biosci..

[12] Tang S., Luo N., Zeng Q., Dong L., Zhang R., He S., Nag A., Huang F., Su D. (2023). Lychee pulp phenolics fermented by mixed lactic acid bacteria strains promote the metabolism of human gut microbiota fermentation in vitro. Food Funct..

[13] Aihaiti A., Zhao L., Maimaitiyiming R., Wang L., Liu R., Mu Y., Chen K., Wang Y. (2025). Changes in volatile flavors during the fermentation of tomato (*Solanum lycopersicum* L.) juice and its storage stabilization. Food Chem..

[14] Zhao Y., Liu R., Mu Y., Lv M., Xing J., Zheng L., Aihaiti A., Wang L. (2024). Study on the mechanisms of flavor compound changes during the lactic fermentation process of peach and apricot mixed juice. Foods.

[15] Lv M., Liu X., Liu R., Aihaiti A., Hong J., Zheng L., Xing J., Cui Y., Wang L. (2025). Untargeted metabolomics reveals flavor and metabolic changes in mixed *Lactobacillus-fermented* black mulberry juice. Food Chem. X.

[16] Liu Y., Sheng J., Li J., Zhang P., Tang F., Shan C. (2022). Influence of lactic acid bacteria on physicochemical indexes, sensory and flavor characteristics of fermented sea buckthorn juice. Food Biosci..

[17] Lv M., Aihaiti A., Liu X., Tuerhong N., Yang J., Chen K., Wang L. (2022). Development of probiotic-fermented black mulberry (*Morus nigra* L.) juice and its antioxidant activity in C2C12 cells. Fermentation.

[18] Pinmanee P., Sompinit K., Jantimaporn A., Khongkow M., Haltrich D., Nimchua T., Sukyai P. (2023). Purification and immobilization of superoxide dismutase obtained from *Saccharomyces cerevisiae* TBRC657 on bacterial cellulose and its protective effect against oxidative damage in fibroblasts. Biomolecules.

[19] Razola-Díaz M.d.C., De Montijo-Prieto S., Guerra-Hernández E.J., Jiménez-Valera M., Ruiz-Bravo A., Gómez-Caravaca A.M., Verardo V. (2024). Fermentation of orange peels by lactic acid bacteria: Impact on phenolic composition and antioxidant activity. Foods.

[20] Zhang X., Xiong K., Ye S., Du M., Wang Z. (2025). Effects of different raw materials on bacterial community and flavor compounds in fermented red sour soup. Food Biosci..

[21] Cai L., Wang W., Tong J., Fang L., He X., Xue Q., Li Y. (2022). Changes of bioactive substances in lactic acid bacteria and yeasts fermented kiwifruit extract during the fermentation. LWT.

[22] Cao H., Bai M., Lou Y., Yang X., Zhao C., Lu K., Zhang P. (2023). Optimization of the brewing process and analysis of antioxidant activity and flavor of elderberry wine. Fermentation.

[23] Luo D., Pang X., Xu X., Bi S., Zhang W., Wu J. (2018). Identification of cooked off-flavor components and analysis of their formation mechanisms in melon juice during thermal processing. J. Agric. Food Chem..

[24] Ao H., Tang C., Lu Y., Zhang Y., He L., Qiu S., Yan Y., Li C. (2025). Characterization of physicochemical properties, sensory characteristics, and volatile compounds with a special focus on the terpene profile of commercial chinese kiwifruit wines. J. Food Compos. Anal..

[25] Huang J., He W., Wang P., Geng J., Zhan P., Tian H. (2024). Characterization of aroma profiles in flat peach juice post-thermal sterilization: Insights from comprehensive two-dimensional GC with time-of-flight mass spectrometry (GC×GC-TOF-MS), sensory analysis, and chemometric approaches. J. Food Compos. Anal..

[26] Chen Y., Huang Y., Bai Y., Fu C., Zhou M., Gao B., Wang C., Li D., Hu Y., Xu N. (2017). Effects of mixed cultures of *Saccharomyces cerevisiae* and *Lactobacillus plantarum* in alcoholic fermentation on the physicochemical and sensory properties of citrus vinegar. LWT.

[27] Lu T., Song B., Yang J., Tan H., Qiao H., Zhi W., Chen R., Sheng Z. (2024). *Lactobacillus* HNC7-YLC92 improves the fermentation quality of cassava–acerola cherry beverage. Fermentation.

[28] Zhang B., Shen J.Y., Wei W.W., Xi W.P., Xu C.J., Ferguson I., Chen K. (2010). Expression of genes associated with aroma formation derived from the fatty acid pathway during peach fruit ripening. J. Agric. Food Chem..

[29] Wu Q., Xu Z., Feng S., Shi X., Qin L., Zeng H. (2024). Correlation analysis between microbial communities and flavor compounds during the post-ripening fermentation of traditional chili bean paste. Foods.

[30] Wu T., Sakamoto M., Phacharapan S., Inoue N., Kamitani Y. (2023). Antioxidant characteristic changes, sensory evaluation, processing and storage of functional water modified juice. Food Biosci..

[31] Morita M., Naito Y., Niki E., Yoshikawa T. (2017). Antioxidant action of fermented grain food supplement: Scavenging of peroxyl radicals and inhibition of plasma lipid oxidation induced by multiple oxidants. Food Chem..

[32] Liu X., Lv M., Maimaitiyiming R., Chen K., Tuerhong N., Yang J., Aihaiti A., Wang L. (2023). Development of fermented sea buckthorn (*Hippophae rhamnoides* L.) juice and investigation of its antioxidant and antimicrobial activity. Front. Nutr..

[33] Lafarga T., Aguiló-Aguayo I., Bobo G., Chung A.V., Tiwari B.K. (2018). Effect of storage on total phenolics, antioxidant capacity, and physicochemical properties of blueberry (*Vaccinium corymbosum* L.) jam. J. Food Process. Preserv..

[34] Pi T.-H., Shiau C.-Y., Chang C.-J., Sung W.-C. (2017). Studies on the development and quality of fish chiffon cake and its storage stability. J. Aquat. Food Prod. Technol..

